# Evaluation of dose delivery based on deformed CT using a commercial software for lung cancer

**DOI:** 10.1038/s41598-024-65381-x

**Published:** 2024-06-24

**Authors:** Jihong Chen, Kaiqiang Chen, Min OuYang, Guohua Wang, Penggang Bai, Hongqiang You

**Affiliations:** 1 Department of Radiation Oncology, Clinical Oncology School of Fujian Medical University, Fujian Cancer Hospital, Fuzhou, 350014 Fujian China; 2 School of Nuclear Science and Technology, University of South China, Hengyang, 421001 China

**Keywords:** Deformable image registration, Lung cancer, CBCT, Adaptive radiotherapy, Lung cancer, Radiotherapy, Biophysics

## Abstract

This study employed a commercial software velocity to perform deformable registration and dose calculation on deformed CT images, aiming to assess the accuracy of dose delivery during the radiotherapy for lung cancers. A total of 20 patients with lung cancer were enrolled in this study. Adaptive CT (ACT) was generated by deformed the planning CT (pCT) to the CBCT of initial radiotherapy fraction, followed by contour propagation and dose recalculation. There was not significant difference between volumes of GTV and CTV calculated from the ACT and pCT. However, significant differences in dice similarity coefficient (DSC) and coverage ratio (CR) between GTV and CTV were observed, with lower values for GTV volumes below 15 cc. The mean differences in dose corresponding to 95% of the GTV, GTV-P, CTV, and CTV-P between ACT and pCT were − 0.32%, 4.52%, 2.17%, and 4.71%, respectively. For the dose corresponding to 99%, the discrepancies were − 0.18%, 8.35%, 1.92%, and 24.96%, respectively. These differences in dose primarily appeared at the edges of the target areas. Notably, a significant enhancement of dose corresponding to 1 cc for spinal cord was observed in ACT, compared with pCT. There was no statistical difference in the mean dose of lungs and heart. In general, for lung cancer patients, anatomical motion may result in both CTV and GTV moving outside the original irradiation region. The dose difference within the original target area was small, but the difference in the planning target area was considerable.

## Introduction

Intensity-modulated radiation therapy (IMRT) has become an important method for the treatment of lung cancer, which generally requires image guidance^1^. Cone-beam computed tomography (CBCT) has emerged as a prevalent onboard imaging modality for linear accelerator machines, typically employed for pre-treatment patient positioning^2,3^. The clinical outcomes of IMRT could be affected by the anatomical variation such as tumor regression and organ movement, due to the sharply dose gradients. It was reported that significant tumor migration was observed for lung cancer patients undergoing radiotherapy^4^. Consequently, adaptive radiation therapy (ART) may be crucial for radiotherapy of lung cancer^5^.

ART relies on the acquisition of 3D imaging data throughout the course of treatment, usually CT, to monitor anatomical variations and enable precise dose calculations^6^. Recently, the image quality of CBCT has improved greatly, such as the iterative CBCT of the Varian company^7^. The integration of routine CBCT images into the process of ART is very attractive. However, the direct dose calculation on CBCT images is prone to significant inaccuracies due to scattering and image artifacts^8,9^.

A number of research and potential techniques have been proposed to resolve this issue^10,11^. The early approach for dose calculation on CBCT was using the planning CT (pCT) calibration curve or CBCT calibration curve, which could bring unstable outcomes^12,13^. It was reported that dose calculation based on a CBCT calibration curve could result in an accuracy of 1–5%^13–15^. An intensive technique is to replace the HU value of CBCT with the HU density or CT numbers of pCT^16–18^. Chen et al. reported that dose calculations for CBCT based on a patient-specific stepwise HU-to-density curve was compared to simulation CT^16^. Then there is the method based on deformable registration, including image deformation and dose deformation^19–22^. It is noteworthy that deformation-based registration approaches exhibit superior promise compared to preceding techniques^23,24^. Deformed CT, which created from pCT to CBCT, mapped accurate HU information from the pCT while keeping anatomical information from CBCT. Veiga et al. studied the calculation accuracy on CBCT images for head and neck patients and discussed the potential implementation of a “dose-driven” online ART, using deformation registration method^19^. Similar studies have been reported to quantify the dosimetric differences between pCT and deformed CT in lung cancer^25^. Valentina et al. demonstrated that deformable registration is an accurate and efficient method for dose calculation on CBCT^26^. Generally, the accuracy of CBCT dose calculation depends on CBCT image quality and accurate CBCT calibration, irrespective of the methods used. The advantage of deformable registration is its speed and the promising results it has shown. However, significant anatomical changes, particularly at tissue-air interfaces, can result in disparities between the CBCT and the deformed images, potentially impacting delineations and dosimetric evaluations^23^. A recently published review summarizes the different methods and associated dose calculation accuracy in the field^10^. However, few of these methods have been widespread applied to routine ART, despite of high dose calculation accuracy.

In this study, we employed a commercially available software velocity to conduct deformable registration and dose calculation on deformed CT images of patients diagnosed with lung cancer, aiming to assess the positional and anatomical displacements between pCT and CBCT and their impact on dose delivery.

## Material and methods

### Image acquisition and processing

Twenty lung cancer patients undergoing radiotherapy at Fujian Cancer Hospital from 2022 to 2024 were included in this study. Ethical approval was obtained from the Ethics Committee of Fujian Cancer Hospital (ethics number: K2022-184-01). All patients provided written informed consent prior to enrollment in the study, while all methods were performed in accordance with the Declaration of Helsinki as well as relevant guidelines and regulations. The specific staging information of these patients was listed in Table 1. All patients underwent a normal CT scans using a Philips large-bore CT scanner (Philips Medical Systems Inc., Cleveland, OH, USA), following a standard thoracic protocol (120 kV, 325 mAs). Patients were positioned supine and immobilized using vacuum cushions and thermoplastic masks. The CT scans were performed with a slice thickness of 5 mm and a pixel size of 512 × 512.

**Table 1:** The characteristics of patients with lung cancer (n = 20).

T stage		N stage		M stage		Overall stage	
T1	7	N0	5	M0	18	Stage I	3
T2	4	N1	0	M1	2	Stage II	1
T3	4	N2	10			Stage III	14
T4	5	N3	5			Stage IV	2
Total	20	Total	20	Total	20	Total	20

The pCT images were transferred to Eclipse (Ver. 15.6, Varian Medical System, Inc.) and reconstructed with a 2 mm slice thickness, where clinician delineated target volumes and organs at risk (OARs). The target volumes included the gross tumor volume (GTV) and clinical target volume (CTV), with expansions of 7 mm to create the GTV-P and CTV-P respectively. The GTV-P represented the planning target volume for GTV, while CTV-P represented that for CTV. The prescribed doses for GTV-P and CTV-P were 61.6 Gy and 50.4 Gy, respectively, delivered in 28 fractions. OARs included the lungs, heart, and spinal cord. A physicist performed volumetric modulated arc therapy (VMAT) planning with Eclipse, using a Truebeam accelerator (Ver. 2.7, Varian Medical System, Inc.). All plans were using 6 MV photon beams, with a pair of coplanar full arcs (178°–182°) with opposite 10° collimator rotation from their neutral position. The dose calculation grid was 2.5 mm while the dose calculation algorithm was Acuros External Beam (Ver. 15.6).

**Figure 1:**
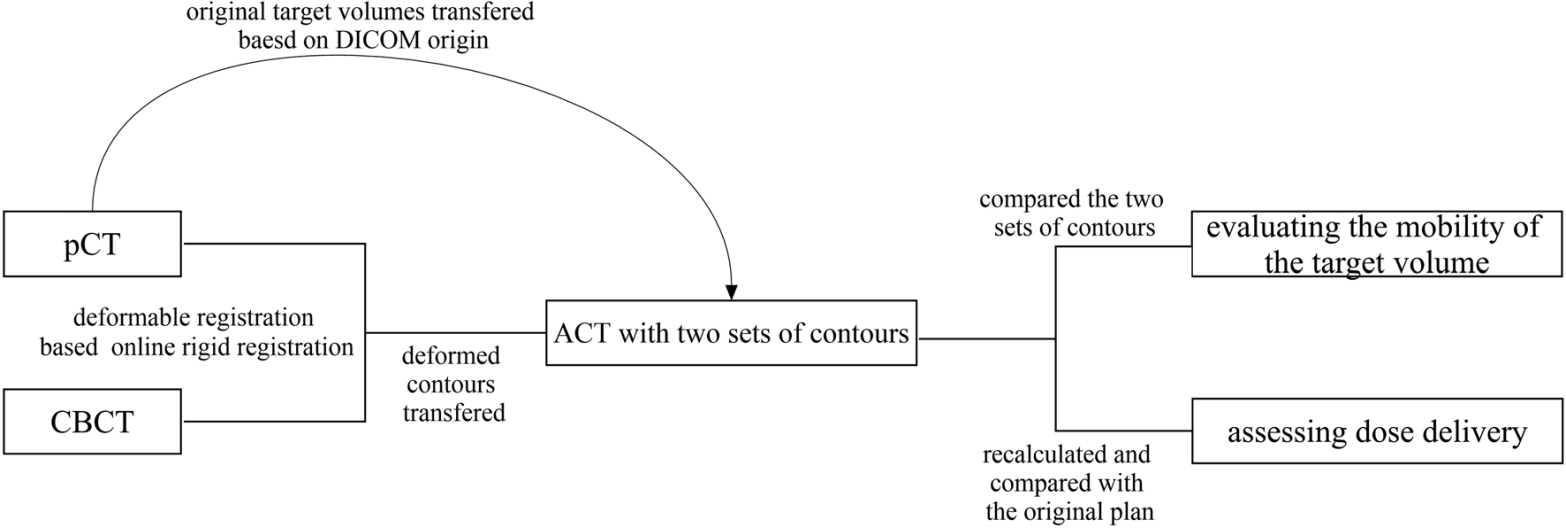
The workflow of image processing and evaluation.

The CBCT images of patients were acquired at the onset of their radiotherapy. The mean time difference between the pCT and CBCT scans was 7.5 ± 1.1 days. The dimension of CBCT image was 512 × 512 pixels with a slice thickness of 2 mm. Subsequently, both CBCT and pCT images were imported into velocity software (Ver. 4.0, Varian Medical System, Inc.) for the generation of adaptive CT (ACT). The process of ACT generation was automatized through a defined workflow, comprising several sequential steps. Initially, deformable registration was conducted between the CBCT and pCT images based on the results of online rigid registration, employing the CBCT corrected multi pass deformable registration algorithm. Following registration, the CT image underwent deformation to align with the CBCT, facilitated by the inverse matrix of the deformation vectors. The contours from the pCT, encompassing target volumes and OARs, were then propagated onto the ACT utilizing the aforementioned deformation vectors. Subsequently, the deformed contours and ACT were transferred to Eclipse, wherein the original target contours were replicated onto the ACT based on the DICOM origin. Consequently, the ACT exhibited both the original and deformed contours of the target volumes concurrently. Finally, the dose distribution of treatment planning was recalculated on the ACT. The flowchart depicting the study process was illustrated in Fig. 1.

### Evaluation for the mobility of target volumes

The volumes of original and deformed targets were utilized for assessing the fidelity of contour propagation for GTV and CTV. Evaluation criteria for the mobility of target volumes included the dice similarity coefficient (DSC) and coverage ratio (CR)^27^, which indicated the coverage ratio of the deformed CTV and GTV areas by the original PTV and GTV-P areas. The corresponding calculation formula is as follows:

${\text{DSC}} = 2\left( {{\text{A}} \cap {\text{B}}} \right)/\left( {{\text{A}} + {\text{B}}} \right)$, where A is the volume deformed CTV or GTV and B is the volume of original CTV or GTV.

${\text{CR}} = \left( {{\text{A}} \cap {\text{B}}} \right)/{\text{A}}$, where A is the volume deformed CTV or GTV and B is the volume of original PTV or GTV-P.

### Evaluation of dose delivery

Several dosimetric parameters were collected to assess variations of dose delivery. The D_95_ and D_99_ (the dose corresponding to 95% and 99% of volume, respectively) for target volumes were calculated. Percentage differences were defined as:

$$ {\Delta D}_{{\text{x}}} {\text{ = [D}}_{{\text{x}}} \left( {{\text{pCT}}} \right) - {\text{D}}_{{\text{x}}} \left( {{\text{ACT}}} \right){\text{]/D}}_{{\text{p}}} , $$

where x is 95 or 99, and D_p_ is a corresponding prescription dose.

Furthermore, V_5_ and V_20_ (the percentage volume receiving 5 Gy and 20 Gy) for lungs, D_mean_ for lungs and heart, and D_1cc_ (the dose corresponding to 1 cc) for spinal cord were calculated.

### Statistical analysis

Paired *t*-tests (for normally distributed data) or Wilcoxon signed-rank tests (for non-normally distributed data) were conducted on these metrics as delineated previously. A one-way ANOVA test was employed to analyze the differences of ∆D_x_ among CTV, CTV-P, GTV and GTV-P. All statistical analyses were performed using statistical package for the social sciences (SPSS 21.0; SPSS Inc., Chicago, IL, USA), with significance set at p < 0.05.

## Results

**Figure 2:**
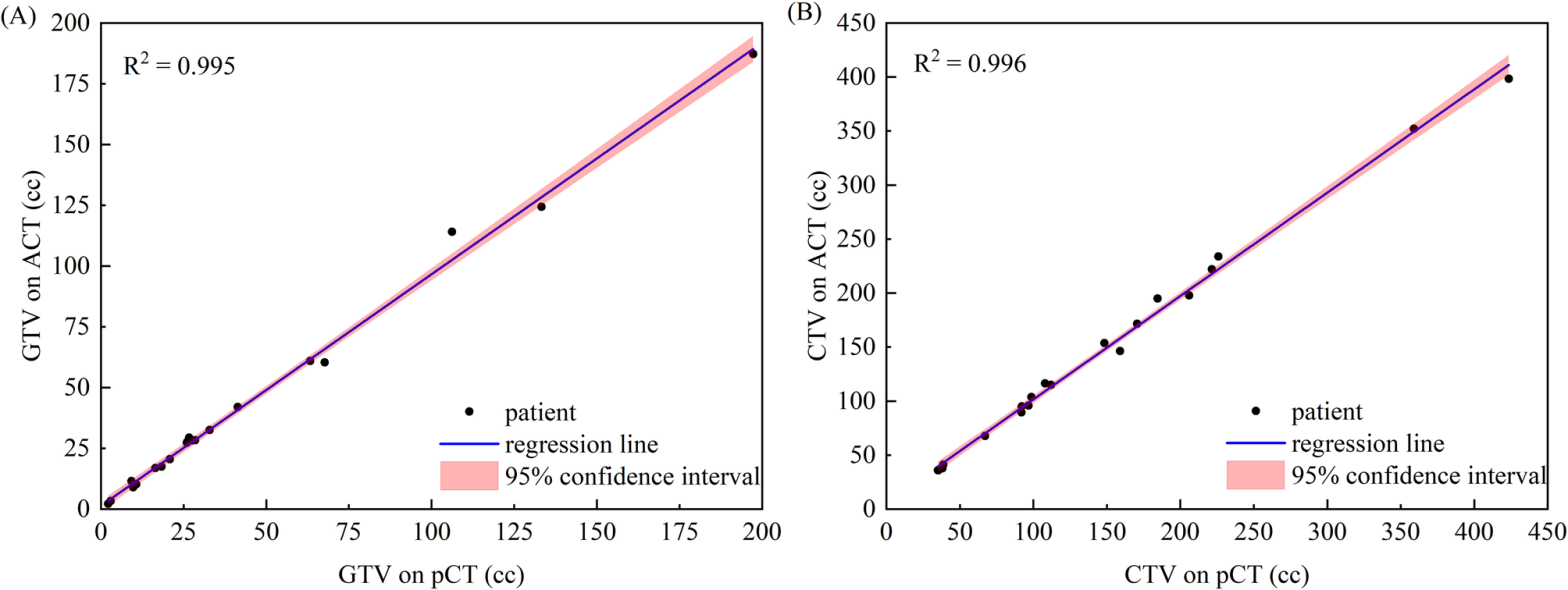
The correlation between volumes obtained from planning CT (pCT) and adaptive CT (ACT). (**A**) For GTV, (**B**) for CTV.

The mean volume of the original GTV was 43.4 cc (range from 2.2 to 197.3 cc), while the deformed GTV volume averaged at 42.7 cc (range from 2.2 to 187.2 cc). Similarly, the mean volume of the original CTV was 145.6 cc (range from 35.1 to 423.5 cc), while the deformed CTV volume averaged at 145.2 cc (range from 35.9 to 398.3 cc). As Fig. 2 shown, statistical analysis revealed no significant differences in volume between the original and deformed contours for either GTV (Wilcoxon signed-rank test, p = 0.825) or CTV (Wilcoxon signed-rank test, p = 0.455).

**Figure 3:**
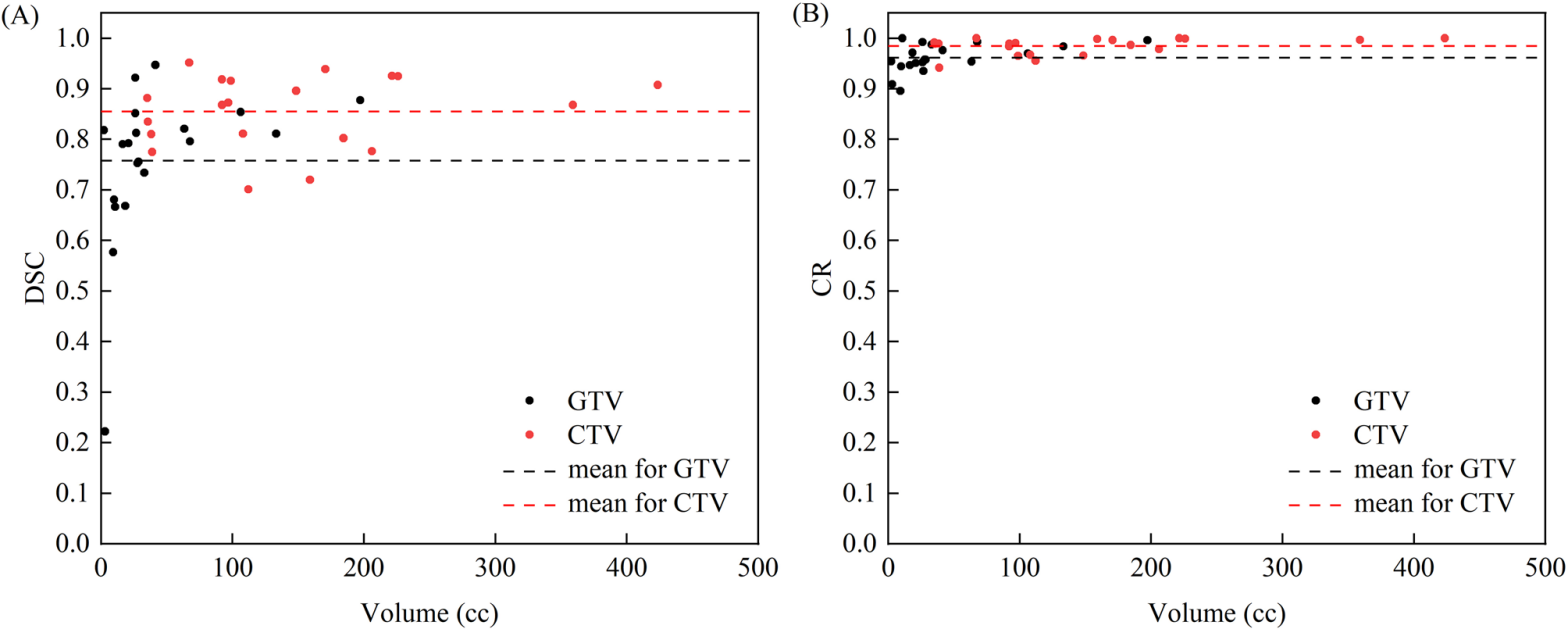
(**A**) The dice similarity coefficient (DSC) to GTV and CTV. (**B**) The coverage ratio (CR) to GTV and CTV.

As Fig. 3 shown, the mean DSC value for GTV was 0.758, while it was 0.855 for CTV. There were statistically significant differences between the DSC for GTV and CTV (paired *t*-test, p = 0.003). Likewise, the mean CR value was 0.962 for GTV and 0.984 for CTV, demonstrating a statistically significant difference (paired *t*-test, p = 0.000). Notably, GTV volumes smaller than 15 cc tended to exhibit lower DSC and CR values.

**Figure 4:**
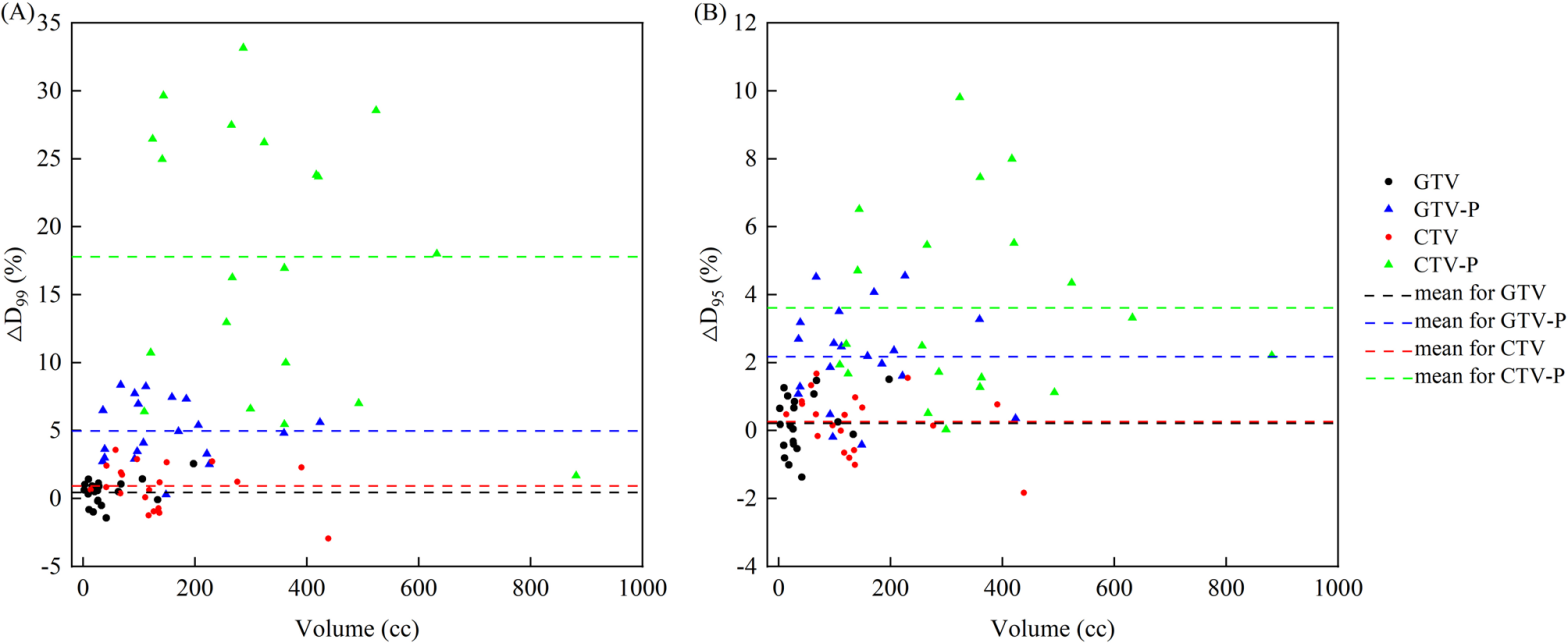
Differences in dosimetric parameters of GTV, GTV-P, CTV and CTV-P between the pCT and ACT images. (**A**) for D_99_, (**B**) for D_95_.

The mean dose differences of D_99_ between pCT and ACT was 0.44% for GTV, 0.91% for CTV, 4.96% for GTV-P and 17.79% for CTV-P, with statistically significant difference (ANOVA test, p = 0.000). Similarly, the mean discrepancy of D_95_ was 0.21% for GTV, 0.29% for CTV, 2.17% for GTV-P and 3.61% for CTV-P, with statistically significant difference (ANOVA test, p = 0.000). As Fig. 4 shown, the differences for GTV fell within ± 2% and CTV fell within ± 4%. It’s noteworthy that the dose difference for CTV-P and GTV-P between pCT and ACT was notably large, particularly for D_99_.

**Figure 5:**
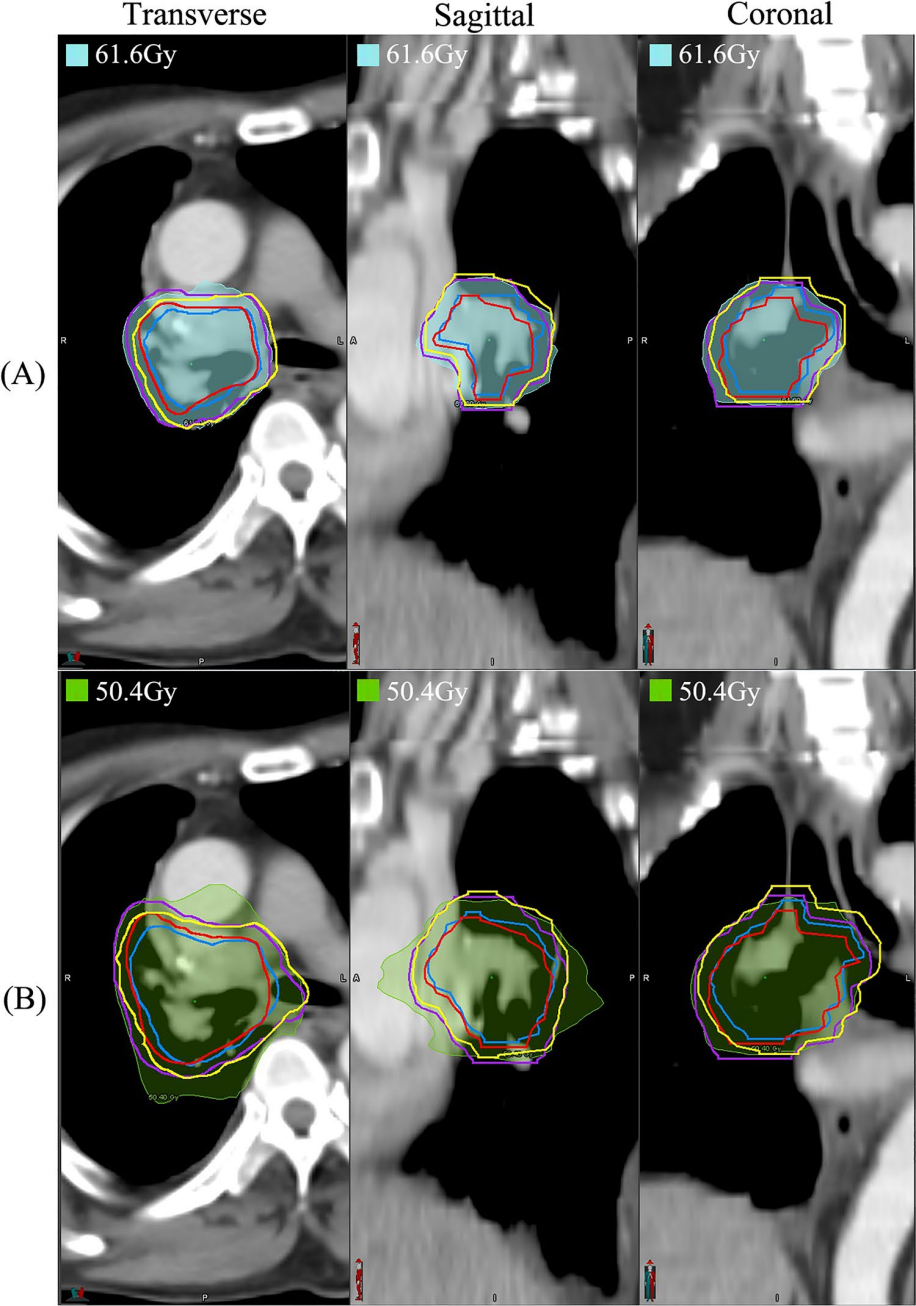
Contours and dose distribution on ACT for a patient. (**A**) The red, blue, purple and yellow lines represent original GTV, deformed GTV, original GTV-P and deformed GTV-P. The shaded areas in light blue represent the prescribed dose line of 61.6 Gy. (**B**) The red, blue, purple and yellow lines represent original CTV, deformed CTV, original CTV-P and deformed CTV-P. The shaded areas in green represent the prescribed dose line of 50.4 Gy.

Figure 5 illustrates the contour comparison and dose distribution on the ACT for one patient. For this patient, the differences in D_95_ for GTV, GTV-P, CTV, and CTV-P were -0.32%, 4.52%, 2.17%, and 4.71%, respectively. For D_99_, the differences were − 0.18%, 8.35%, 1.92%, and 24.96%, respectively. It can be observed that both the GTV and CTV, after deformation, do not exceed the regions of GTV-P and CTV-P. However, GTV-P and CTV-P are deformed beyond their original regions, particularly at the edges. This deformation resulted in lower doses at the edges of CTV-P and GTV-P.

In terms of OARs, as shown in Table 2, the D_mean_ for lungs was 11.49 Gy and 11.59 Gy, in pCT and ACT respectively, with no statistically significant difference (paired *t*-test, p = 0.172). There was also no statistical difference for D_mean_ of heart (11.81 Gy vs 11.55 Gy, Wilcoxon signed-rank test, p = 0.117). However, the D_1cc_ for the spinal cord exhibited a statistically significant difference, with values of 37.52 Gy and 38.12 Gy, respectively (paired *t*-test, p = 0.026).

**Table 2:** Differences in dosimetric parameters for the OARs between pCT and ACT (mean ± SD).

OARs	Index	pCT	ACT	p
Lungs	D_mean_ (Gy)	11.49 ± 3.06	11.59 ± 3.04	0.172^#^
	V_20_ (%)	20.22 ± 6.11	20.40 ± 6.08	0.291^#^
	V_5_ (%)	49.90 ± 12.31	50.03 ± 12.21	0.465^#^
Heart	D_mean_ (Gy)	11.81 ± 11.17	11.55 ± 11.46	0.117*
Spinal cord	D_1cc_ (Gy)	37.52 ± 8.00	38.12 ± 8.60	0.026^#^

*represented Wilcoxon signed-rank test.

^#^represented paired *t*-test.

## Discussion

In this study, the commercial software velocity was employed to investigate anatomical variation and assess the accuracy of the actual delivered dose in radiotherapy for lung cancer. ACT was generated by deforming the pCT to CBCT, with both deformed and original contours transferred onto the ACT for dose recalculations. Theoretically, dose conversions of the original treatment plan on ACT yield smaller errors compared to direct calculations on CBCT. Results indicate significantly higher DSC values for CTV compared to GTV, particularly evident with larger volumes. Dose discrepancies for GTV and CTV within 4% were observed in the initial treatment of 20 lung cancer patients, with average discrepancies remaining below 1%. However, the average dose discrepancies for CTV-P and GTV-P were substantial, with the D_99_ of CTV-P reaching 17.79%. Except for the spinal cord, significant differences in the dose received by other OARs were not detected.

The volume of anatomical structures significantly influences the outcomes of registration, subsequently affecting the accuracy of contour propagation^27,28^. Notably, the DSC of GTV decreases when the target volume is less than 15 cc, ranged from 0.22 to 0.81. Conversely, due to the larger volumes associated with the CTV, DSC values are relatively higher, with all but two cases exceeding 0.75. It is noteworthy that volumetric enlargement does not invariably correlate with increased DSC values, due to the mobility of tumors. For instance, when the volume of GTV surpasses 60 cc, its DSC predominantly remains at 0.8, while the volume of CTV exceeds 220 cc, the DSC remains around 0.9. Meanwhile, neither CTV nor GTV consistently achieves a CR value of 1, suggesting the potential for CTV and GTV to extend beyond the planning target volume (CTV-P and GTV-P) boundaries due to tumor motion. Previous studies employing 4DCT for internal target volume (ITV) delineation and subsequent expansion to PTV have demonstrated CR values approaching 1, attributed to the accurate capture of lung tumor motion range^27,29^.

From a dosimetric standpoint, both CTV and GTV exhibit minimal dose discrepancies, generally below 3% and predominantly within 2%, which are clinically acceptable. The D_99_ difference in GTV was greater than 2% in only one patient, likely due to its less uniform dose distribution, though it still exceeded the prescribed dose. In contrast, the difference in D_99_ of CTV exceeded 2% in several patients. This may be because, to preserve lung function, the prescribed dose coverage of CTV-P did not reach 100%, resulting in significant dose variation in the marginal area. However, it is essential to note that these findings pertain solely to the initial treatment fraction. The dose discrepancies may increase with the treatment progresses, due to tumor regression or motion dynamics. Widely adopted strategies such as expanding the irradiation field of target areas, typically by 7 mm in this study, may mitigate such deviations. In addition, although clinicians may prefer to focus on the actual exposure doses of GTV and CTV, the doses of their corresponding PTVs can vary greatly, particularly the D_99_ of CTV-P. The main reason for this variation is the change in the upper and lower bounds of the target area. Due to the steep drop in the dose gradient of the intensity modulation plan, especially with the limitations of the JAW in the cranial and caudal directions, the displacement of one or two layers can cause significant dose changes. This issue needs to be carefully considered in clinical practice. Moreover, it is worth noting that the dose to the spinal cord may surpass planned levels, necessitating clinical consideration.

Previous investigations focusing on deformable registration for assessing lung cancer radiation dose accuracy typically emphasized PTV variations^30,31^. For instance, Disher et al. reported that the D_95_ of PTV for lung cancer in the corrected CBCT differed by − 4% to 9%, compared to that in the pCT^32^. Giacometti et al. reported a median difference of − 0.3% for D_99_ of PTV, between deformed CT and pCT^26^. Similarly, a mean dose metric error less than 1% could be achieved by deformable registration algorithms for lung cancer, as Marchant et al. indicated^23^. Cole et al. showed that the dose distributions on the deformed CT and replan CT matched closely^21^. And for stereotactic radiotherapy of lung cancer, differences between D_99_ of the ITV on pCT and rCT could reach ± 10%, due to inhomogeneous dose distributions^27^. In this study, the dose delivery accuracy of CTV, GTV, CTV-P and GTV-P was evaluated simultaneously. The dose difference of GTV and CTV was relatively small, primarily because most of their volume remained within their respective PTV zones. However, the dose discrepancies of GTV-P and CTV-P in this study were larger than those reported in previous studies. This could be attributed to various factors such as the deformation algorithm of commercial software, scanning conditions, time intervals between CT and CBCT scans, and treatment plan details. For lung cancer patients, due to the movement and deformation of the organ and the tumor, the actual radiation target may not perfectly align with the planning target. Consequently, some target areas’ edges may extend beyond the irradiated area, necessitating the expansion of the target area. Even slight changes in the shape and displacement of the patient can significantly impact the dose distribution, particularly at the edges of the exposure area where dose reduction is steep.

Currently, an increasing number of studies are adopting deep learning methodologies to generate pseudo-CT images from CBCT, with quality and HU values comparable to CT images^33–35^. However, deep learning models were usually difficult to be integrated with existing radiotherapy equipment. It demands significant computational expertise and a substantial training dataset. Utilizing commercial software remains a more clinically suitable and practical approach.

The limitations of this study include the relatively limited number of cases, although higher than those in current literature (typically below 15 cases). Additionally, only outcomes from the initial treatment were considered, with potential for greater tumor regression and dose variations in subsequent fractions. Future endeavors should focus on increasing case numbers and utilizing ACT for dose calculations throughout all treatment fractions.

## Conclusion

The primary objective of this paper is to utilize CBCT and deformable registration methods to investigate positional and anatomical displacement in lung cancer patients and its impact on dose delivery.

This study employed commercial software velocity to generate deformed CT images from CBCT for lung cancer, to investigate the variations in target area position and accuracy of dose delivery during radiotherapy. For lung cancer patients, anatomical variation may result in both CTV and GTV moving outside the original irradiation region. It was found that the dose difference within the original target area was minor, whereas the disparity in the PTV target area was significant.

## Data Availability

The datasets used and/or analysed during the current study available from the corresponding author on reasonable request.
